# Targeting RNA for processing or destruction by the eukaryotic RNA exosome and its cofactors

**DOI:** 10.1101/gad.294769.116

**Published:** 2017-01-15

**Authors:** John C. Zinder, Christopher D. Lima

**Affiliations:** 1 Tri-Institutional Training Program in Chemical Biology, Memorial Sloan Kettering Cancer Center, New York, New York 10065, USA;; 2 Structural Biology Program, Sloan Kettering Institute, New York, New York, 10065, USA;; 3 Howard Hughes Medical Institute, New York, New York, 10065 USA

**Keywords:** 3′ to 5′, RNA degradation, RNA exosome, RNA processing, endoribonuclease, exoribonuclease, exosome

## Abstract

The eukaryotic RNA exosome is an essential and conserved protein complex that can degrade or process RNA substrates in the 3′-to-5′ direction. Since its discovery nearly two decades ago, studies have focused on determining how the exosome, along with associated cofactors, achieves the demanding task of targeting particular RNAs for degradation and/or processing in both the nucleus and cytoplasm. In this review, we highlight recent advances that have illuminated roles for the RNA exosome and its cofactors in specific biological pathways, alongside studies that attempted to dissect these activities through structural and biochemical characterization of nuclear and cytoplasmic RNA exosome complexes.

## Abstract (precis)

In this review, Zinder and Lima highlight recent advances that have illuminated roles for the RNA exosome and its cofactors in specific biological pathways, alongside studies that attempted to dissect these activities through structural and biochemical characterization of nuclear and cytoplasmic RNA exosome complexes.

The eukaryotic RNA exosome is a conserved multisubunit protein complex that catalyzes 3′-to-5′ processing or degradation of a vast array of different RNA substrates (Januszyk and Lima 2014; Kilchert et al. 2016). Since its discovery as a key factor involved in 3′ processing of ribosomal RNAs (rRNAs) during ribosome biogenesis in budding yeast (Mitchell et al. 1997), transcriptome-wide analyses in diverse eukaryotic model systems revealed that the RNA exosome contributes to the processing and/or degradation of nearly every class of RNA (Chekanova et al. 2007; Gudipati et al. 2012; Schneider et al. 2012; Pefanis et al. 2014).

Nuclear and cytoplasmic forms of the RNA exosome are defined by unique subunit compositions that interact with distinct cofactors in these subcellular compartments (Table 1). In the cytoplasm of *Saccharomyces cerevisiae*, the exosome includes a nine-subunit core (Exo9) that interacts with Dis3 to form a 10-subunit complex (Exo10^Dis3^). The Exo9 core lacks catalytic activity, while Dis3 catalyzes endoribonuclease (endo) and processive 3′-to-5′ exoribonuclease (exo) activities (Liu et al. 2006; Dziembowski et al. 2007; Lebreton et al. 2008). Although redundant with cytoplasmic 5′-to-3′ decay pathways (Anderson and Parker 1998), Exo10^Dis3^ contributes to translation-dependent mRNA surveillance pathways such as nonstop decay (NSD), nonsense-mediated decay (NMD), and no-go decay (NGD) (for review, see Łabno et al. 2016). All 10 genes encoding subunits of Exo10^Dis3^ are essential for viability in yeast (Mitchell et al. 1997; Brouwer et al. 2000). While *dis3* alleles that disrupt its endo activity bear few phenotypic defects, mutations that disrupt its exo activity result in slow growth, and mutations that disrupt both activities result in synthetic growth defects or inviability (Lebreton et al. 2008). In the nucleus, Exo10^Dis3^ associates with a distributive 3′-to-5′ exoribonuclease Rrp6 and its obligate binding partner, C1D, to form a 12-component complex (Exo12^Dis3/Rrp6/C1D^) (Allmang et al. 1999b; Feigenbutz et al. 2013). While Rrp6 is not essential, Δ*rrp6* strains display a slow growth phenotype, temperature sensitivity, and RNA processing defects (Briggs et al. 1998; Allmang et al. 1999a,b).

**Table 1.**
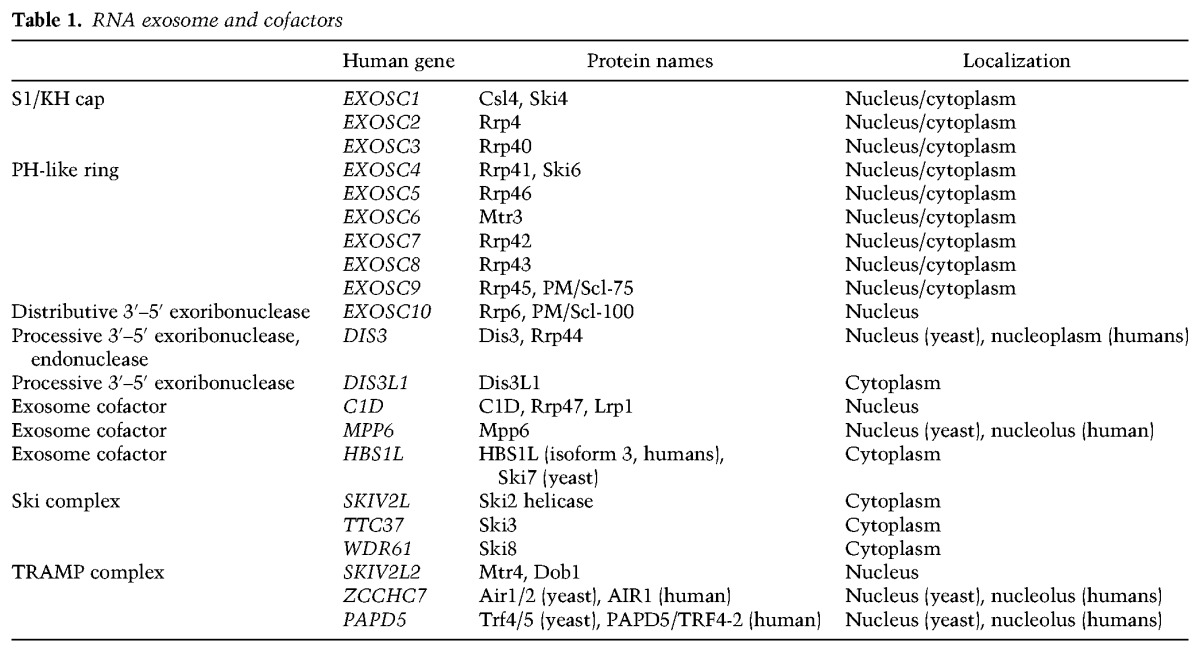
RNA exosome and cofactors

Subunit compositions of nuclear and cytoplasmic RNA exosomes from humans resemble yeast, with some notable differences. For instance, humans encode two exosome-associated Dis3 enzymes, DIS3 and DIS3L, that localize to the nucleus and cytoplasm, respectively (Staals et al. 2010; Tomecki et al. 2010). Similar to yeast, human DIS3 and DIS3L catalyze processive exo activity, although only DIS3 has an intact endonuclease site. DIS3 is excluded from the nucleolus in human cells, while RRP6 is localized to the nucleus and enriched in the nucleolus (Targoff and Reichlin 1985; Staals et al. 2010; Tomecki et al. 2010), suggesting that the nuclear RNA exosome in humans includes Exo9, DIS3, and RRP6/C1D (Exo12^DIS3/RRP6/C1D^) and that a nucleolar exosome may include Exo9 and RRP6 as the only nuclease, presumably associated with C1D (Exo11^RRP6/C1D^). Mammalian cells lacking DIS3 cannot grow, and mutations that disrupt both DIS3 exo and endo activities are synthetic-lethal in HeLa cells, indicating that DIS3 activities are not redundant with other RNA decay pathways (Tomecki et al. 2014).

This review focuses on recent developments pertaining to the diverse biological functions of the exosome and our current understanding of how its structure and biochemical activities enable it to achieve these functions. This will include a brief survey of newly uncovered biological roles for the RNA exosome as well as an overview of our current knowledge for the structural basis of interactions between exosomes, RNA substrates, and cofactors that influence its processing and/or degradation activities.

## The RNA exosome and its roles in cellular homeostasis

Because the RNA exosome is essential for viability in single-celled organisms, it is perhaps unsurprising that it contributes to important and diverse biological processes in higher eukaryotes and is mutated in several diseases (for review, see Staals and Pruijn 2010; Fabre and Badens 2014; Robinson et al. 2015). Here we review recent advances in our understanding of how the exosome and its cofactors contribute to proliferation, differentiation, innate immunity against RNA viruses, and telomerase activity.

### Proliferation and differentiation

Dis3 has gained notoriety for its role in cellular proliferation and was identified as one of the most highly mutated genes in genome-wide association studies of multiple myeloma (MM) (Chapman et al. 2011; Walker et al. 2012; Lohr et al. 2014). Most mutations observed in these studies cluster within its exoribonuclease domain and are predicted to disrupt its 3′-to-5′ decay activity. As Dis3 activities are generally associated with promoting cell division (Ohkura et al. 1988; Tomecki et al. 2014), inactivation of Dis3 in MM was somewhat perplexing. A recent study addressed this by characterizing inactivating mutations of Dis3 in *Drosophila*, *Caenorhabditis elegans*, and mouse models (Snee et al. 2016). While mutating Dis3 alone resulted in mitotic defects, increased RAS activities acted synergistically with this mutant to stimulate growth, a phenotype that was not evident using activated RAS alone. This perhaps explains the observation that RAS activities were often increased in MM clones that contained inactivating Dis3 mutations (Lohr et al. 2014). Furthermore, another study depleted Dis3 in human MM and other cell lines and observed accumulation of Let28Bp, a protein that sequesters the *let-7* family of microRNAs (miRNAs) to prevent their maturation (Segalla et al. 2015). Because *let-7* RNAs can silence *MYC*, *RAS*, and other mRNAs, Dis3 depletion ultimately results in accumulation of these gene products, potentially explaining correlations observed for Dis3 inactivation and RAS activation in model systems (Snee et al. 2016) and MM (Lohr et al. 2014). Perhaps consistent with this model, decreased Dis3 expression has been observed for high-risk genotypes associated with pancreatic cancer (Hoskins et al. 2016), where activating RAS mutants are common (Eser et al. 2014).

Recent work has also illuminated a role for the exosome during erythropoiesis, the process through which hematopoietic stem cells differentiate into erythrocytes (McIver et al. 2014, 2016). In this process, the balance between hematopoietic stem cell differentiation and proliferation is critical: Proliferation can lead to tumor formation, while differentiation can exhaust the supply of stem cells. For erythropoiesis, proliferation and terminal differentiation are enforced by stem cell factor (SCF) and erythropoietin, respectively. GATA-1 and Foxo-3 are master transcription factors that control differentiation during erythropoiesis, and both down-regulate expression of Exosc8, an Exo9 subunit (Table 1). Interestingly, shRNA knockdown of exosome core subunits in hematopoietic stem cells resulted in an accumulation of GATA-1- and Foxo-3-regulated transcripts, suggesting that the exosome may counter differentiation by degrading these transcripts in the absence of erythropoietin (McIver et al. 2014), similar to a role proposed for the exosome in maintaining a proliferative state in human skin stem cells via selective targeting of the *GRLH* mRNA (Mistry et al. 2012). Furthermore, hematopoietic stem cells depleted of exosome components were nonresponsive to SCF due to decreased levels of its cognate receptor tyrosine kinase, Kit, although they remained responsive to erythropoietin (McIver et al. 2016).

### Viral defense

A role for the exosome and its cofactors in viral defense was described nearly two decades before its discovery through a genetic screen that identified the “*SKI*” genes in *S. cerevisiae*. These genes were so named because of the “superkiller” phenotype observed: Mutations in *SKI* genes increased levels of a killer toxin that was produced by the M viral dsRNA (Toh-E et al. 1978; Ridley et al. 1984). It was later discovered that three of these proteins (Ski2, Ski3, and Ski8) form the Ski complex (Brown et al. 2000) that interacts with the RNA exosome via another protein, Ski7 (van Hoof et al. 2000; Araki et al. 2001). Other *SKI* genes were later identified as subunits of the exosome itself (Table 1).

A more recent study using cultured human cells revealed a role for the Ski complex in antiviral defense against hepatitis B virus (HBV) (Aly et al. 2016). A screen for helicases that could suppress HBV replication identified the human Ski complex RNA helicase SKIV2L (Table 1). They further demonstrated that interactions between the HBV X-RNA, SKIV2L, HBS1L (recently identified as the human Ski7 homolog), and the exosome resulted in selective degradation of the HBV X-RNA via the NSD pathway.

The Trf–Air–Mtr4 polyadenylation (TRAMP) complex is a set of cofactors that prepares RNA substrates for degradation by the nuclear RNA exosome (see below; Table 1; LaCava et al. 2005; Wyers et al. 2005); however, this complex was recently shown to participate in viral defense in the cytoplasm (Molleston et al. 2016). Infection of human and *Drosophila* cells with the disparate RNA viruses vesicular stomatitis virus, Sindbis virus, or Rift Valley fever virus (RVFV) was potentiated by knockdown of exosome and TRAMP components. While normally restricted to the nucleus (Lubas et al. 2011), subunits of the TRAMP complex are exported to the cytoplasm during infection, where they participate in the degradation of viral RNAs (Molleston et al. 2016). Furthermore, appending a 3′ untranslated region (UTR) from RVFV to a GFP reporter was sufficient to stimulate its degradation upon RVFV infection in human cells, suggesting that viral RNAs are targeted for selective degradation under these conditions. The mechanisms underlying TRAMP export to the cytoplasm and targeting of viral 3′ UTRs await further investigation.

### Telomerase RNA (hTR) quality control

Several recent studies implicated the exosome and its cofactors in degradation and quality control of hTR in HeLa cells (Nguyen et al. 2015a; Tseng et al. 2015; Shukla et al. 2016). Degradation of hTR is stimulated by the 3′ polyadenylation activity of the human TRAMP complex and antagonized by the poly(A)-binding protein PABPN1 and the deadenylase PARN, which is mutated in some cases of the premature aging disease dyskeratosis congenita (DKC). One study further showed that knockdown of nuclear RNA decay machinery could rescue hTR levels and defects in telomerase activity in cells depleted of dyskerin, a protein subunit of the telomerase RNP that is also mutated in DKC, prompting the investigators to suggest that the exosome could be a therapeutic target for certain telomere pathologies (Shukla et al. 2016).

DGCR8, a dsRNA-binding protein involved in miRNA biogenesis, has been implicated recently as an adaptor protein for exosome targeting to structured substrates such as hTR (Macias et al. 2015). DGCR8 contains dsRNA-binding and heme domains, which interact with the stem and apical regions of the pri-miRNA, respectively, as a dimer (Nguyen et al. 2015b). In the nucleoplasm, this dimer interacts with DROSHA, an RNA endonuclease involved in miRNA maturation, to ensure its fidelity in producing miRNAs (for review, see Macias et al. 2013). Investigators found that DGCR8 also interacts in a distinct complex with the nucleolar exosome, and this interaction is necessary for the turnover of snoRNAs and hTR in that compartment (Macias et al. 2015).

## RNA exosome structure and activities and RNA paths to enzymatic subunits

While the structure of the human Exo9 core was obtained more than a decade ago, more recent crystal and cryo-electron microscopy (cryo-EM) structures have revealed architectures for intact yeast cytoplasmic and nuclear RNA exosomes in complex with RNA substrates and cofactors. Combined with biochemical and genetic studies, these structures illuminate roles for the noncatalytic core in modulating the activities of the associated ribonucleases and the impact of RNA path selection with respect to the fate of RNA substrates.

### RNA exosome core and catalytic subunits

**Figure 1.**
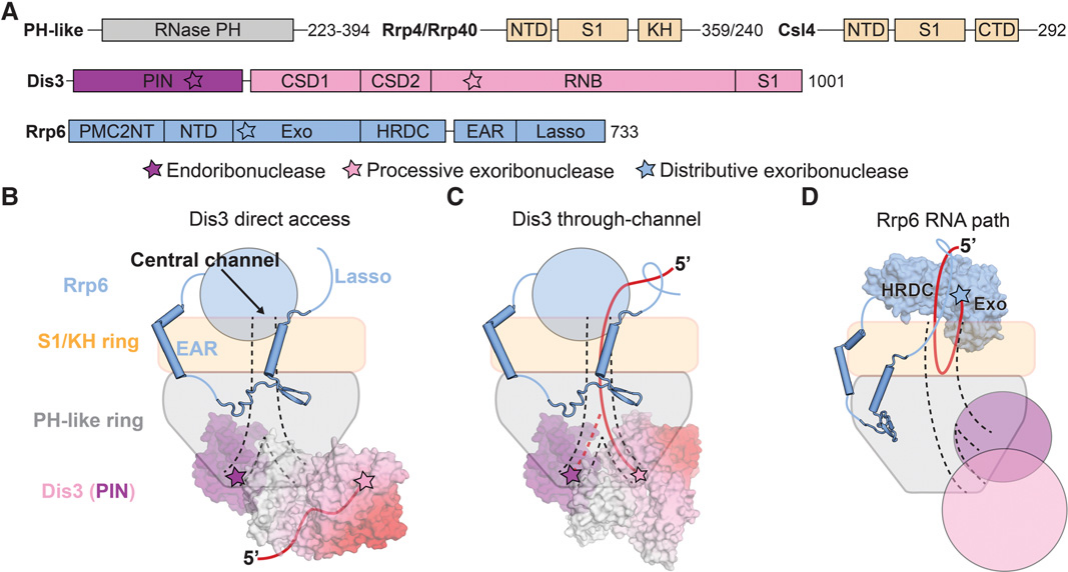
RNA paths and Dis3 conformations in the yeast nuclear exosome. (*A*) Domain schematics for *S. cerevisiae* nuclear exosome components. Catalytic sites are represented by stars, and amino acid lengths for the *S. cerevisiae* proteins are indicated. (*B*) Direct access conformation of Dis3. Dis3 and the Rrp6 exosome-associated region (EAR) domain are from Protein Data Bank (PDB) 5K36. The central channel is indicated by black dashed lines, including a speculative path to the Dis3 endonuclease site, and RNA is represented as a red line with the 5′ end indicated. (*C*) Through-channel conformation of Dis3. Dis3 is from PDB 4IFD, and the Rrp6 EAR domain is from PDB 5K36. RNA and the central channel are indicated as previously, with the dashed red line representing a speculative RNA path to the Dis3 endo active site. (*D*) Rrp6 catalytic module (from PDB 5K36) bound to the core with RNA in its active site. The RNA path to Rrp6 is based on biochemical data and PDB 5K36.

The architecture of the exosome core and its similarity to bacterial and archaeal RNases have been reviewed recently (Januszyk and Lima 2014). Briefly, the Exo9 core includes a hexameric ring of six RNase PH-like domain-containing proteins (Rrp41, Rrp42, Rrp43, Rrp45, Rrp46, and Mtr3; the PH-like ring). This ring is capped by a ring of three proteins that are collectively termed the S1/KH cap because two proteins harbor an N-terminal domain (NTD), S1 domain, and KH domain (Rrp4 and Rrp40), and the third includes the NTD, S1 domain, and C-terminal domain (CTD; Csl4). Together, Exo9 forms a noncatalytic doughnut-shaped complex with a prominent central channel that is wide enough to accommodate ssRNA (Fig. 1; Table 1; Liu et al. 2006).

Catalytic subunits of the RNA exosome include Rrp6 and at least one isoform of Dis3. Dis3 and Dis3L (in the cytoplasm of higher eukaryotes) include an active site that catalyzes processive Mg^2+^-dependent hydrolytic 3′-to-5′ exoribonuclease activity (Dziembowski et al. 2007; Staals et al. 2010; Tomecki et al. 2010; Makino et al. 2013). Dis3 from yeast and humans includes a second active site in the PilT N-terminal (PIN) domain that catalyzes distributive Zn^2+^/Mn^2+^-dependent endoribonuclease activity (Lebreton et al. 2008; Schneider et al. 2008; Schaeffer et al. 2009). Dis3 enzymes associate with exosome PH-like ring subunits opposite to their surfaces that interact with the S1/KH cap (Fig. 1; Bonneau et al. 2009). The nuclear subunit Rrp6 includes a single active site that catalyzes Mg^2+^-dependent distributive 3′-to-5′ hydrolytic exoribonuclease activity (Targoff and Reichlin 1985; Burkard and Butler 2000). The catalytic domain of yeast Rrp6 rests atop the Exo9 core using surfaces opposite to that used by Dis3 (Fig. 1; Wasmuth et al. 2014).

An early hypothesis proposed that RNA degradation by the exosome was conceptually similar to protein degradation by the proteasome (van Hoof and Parker 1999). In this model, the respective active sites are sequestered from the cellular milieu to prevent spurious degradation, and purposeful degradation requires that substrates be licensed to gain access to the active sites through a restricted channel. Subsequent biochemical, structural, and genetic studies largely confirmed this hypothesis, including the observation that Rrp6 and Dis3 activities were modulated or inhibited when associated with the Exo9 core (Dziembowski et al. 2007; Bonneau et al. 2009; Wasmuth and Lima 2012) and that various surfaces within the Exo9 central channel were important for guiding RNA to the respective active sites (Wasmuth and Lima 2012, 2017; Drazkowska et al. 2013; Wasmuth et al. 2014). Subsequent structures also support these models. RNA can thread through the Exo9 central channel to reach Dis3 for processive degradation (Makino et al. 2013; Kowalinski et al. 2016; Liu et al. 2016) or can be deflected back to the Rrp6 active site for distributive processing or degradation (see below; Fig. 1C,D; Wasmuth et al. 2014; Zinder et al. 2016).

### Yeast Dis3 conformations and RNA paths

Structures of *S. cerevisiae* RNA exosome complexes with Dis3 revealed two prominent conformations for the enzyme (Bonneau et al. 2009; Makino et al. 2013, 2015; Liu et al. 2014, 2016; Zinder et al. 2016). While the PIN domain, which contacts the PH-like proteins Rrp41 and Rrp45, remains static, the exoribonuclease module rotates nearly 120° between the two conformations (Fig. 1B,C). One conformation can bind short ssRNAs (<14 nucleotides [nt]), bypassing the Exo9 central channel (termed the direct access Dis3 conformation) (Fig. 1B). This conformation features an extensive interaction surface with Exo9 and is also observed in the absence of RNA, suggesting that it is the resting state of the RNA exosome (Bonneau et al. 2009; Liu et al. 2014, 2016). The other conformation is observed when Dis3 binds longer RNAs (>24 nt) that can span the Exo9 central channel (termed the through-channel conformation) (Fig. 1C; Makino et al. 2013; Liu et al. 2014). This conformation features fewer interactions with Exo9 and is thought to be stabilized by the presence of long RNAs (Liu et al. 2016). Additionally, biochemical data support a through-channel RNA path to the Dis3 endonuclease site, although this model lacks structural confirmation (Wasmuth and Lima 2012; Drazkowska et al. 2013).

The potential importance for these two Dis3 conformations was underscored by two recent studies (Han and van Hoof 2016; Zinder et al. 2016). In one case, investigators analyzed the in vivo effects in a strain expressing a *dis3* allele that contained point mutations predicted to destabilize the direct access Dis3 conformation (Han and van Hoof 2016). Among many observations, they showed that cells expressing this *dis3* allele were viable, albeit with a slow growth phenotype and defects in the degradation of structured substrates. Perhaps most intriguing was the observation that this *dis3* allele suppressed the growth defects/lethality of mutations designed to occlude the Exo9 central channel (Wasmuth and Lima 2012), suggesting strong genetic interactions between mutations in the Exo9 core and Dis3. In the second study, the in vitro effects for mutations within Exo9 subunits predicted to disrupt contacts to Dis3 in both conformations were analyzed (Zinder et al. 2016). Deletions of regions of Rrp45 and Rrp43 proteins that interacted with the direct access Dis3 conformation resulted in reconstituted exosome complexes with increased Dis3 activity. Conversely, deleting a portion of Rrp43 that interacted with the through-channel Dis3 conformation resulted in a measurable decrease in activity. Furthermore, deletion of both Rrp43 elements suppressed the effects of either, suggesting that Exo9 contacts to Dis3 can modulate its activities by stabilizing one or the other Dis3 conformation. The elements in the Exo9 core that make conformation-specific contacts to Dis3 are unique to budding yeast proteins, so it remains unclear whether other eukaryotic exosomes modulate Dis3 activities by similar means.

### Rrp6 conformations and RNA paths

Rrp6 is tethered to the Exo9 core through a C-terminal exosome-associated region (EAR) that wraps around the S1/KH cap and PH-like ring (Fig. 1). In several structures, its catalytic domain is positioned atop the Exo9 core via interactions between the Rrp6 helicase and RNase D CTD (HRDC) and Exo domain and a conserved surface on the S1/KH ring near the entrance to the central channel (Fig. 1D; Wasmuth et al. 2014; Makino et al. 2015; Zinder et al. 2016). However, the catalytic module of Rrp6 can be displaced at equilibrium if a structured RNA with a 3′ overhang long enough to reach a catalytically inactivated Dis3 is present (Makino et al. 2015).

A model in which RNA accesses the Rrp6 catalytic site via interactions with the S1/KH ring proteins is supported by UV cross-linking, biochemical analysis of complexes with mutant S1/KH or Rrp6 subunits (Wasmuth and Lima 2012, 2017), and two recent structures. These structures show the 3′ end of RNA anchored to the Rrp6 active site, with the RNA path directed toward the S1/KH region of the central channel. While the remaining RNA was disordered in an earlier structure (Wasmuth et al. 2014), a more recent model showed that RNA can be deflected by the S1/KH ring to position its 5′ end near a channel formed between the HRDC and Exo domains of Rrp6 (Fig. 1D; Zinder et al. 2016).

While some models suggest that Rrp6 plays a passive role during Dis3-mediated RNA decay (Makino et al. 2015), other data suggest that Rrp6 can enhance Dis3 activities in the nuclear RNA exosome. One line of evidence supporting this is that degradation of poly(A)^+^ transcripts that accumulate in yeast strains lacking Rrp6 can be partially rescued by expressing catalytically inert Rrp6 (Assenholt et al. 2008; Mukherjee et al. 2016). Furthermore, in vitro studies showed that Rrp6 protein can activate the RNA decay activities of Dis3, especially evident for substrates with poly(A) tails (Wasmuth and Lima 2012, 2017; Wasmuth et al. 2014). Interestingly, Dis3 activation requires two domains of Rrp6, its catalytic Exo/HRDC module and its C-terminal tail, termed the RNA lasso (Fig. 1; Wasmuth and Lima 2017). While the catalytic domain binds the S1/KH ring to presumably widen the central channel, the C-terminal domain binds RNA to enhance Rrp6 and Dis3 activities on a variety of RNA substrates. Although disordered in all available structures, it is perhaps noteworthy that the RNA lasso is positioned near the top of the Exo9 core, where it could assist binding RNA adjacent to the central channel (Fig. 1B–D).

## TRAMP and Ski complexes

**Figure 2.**
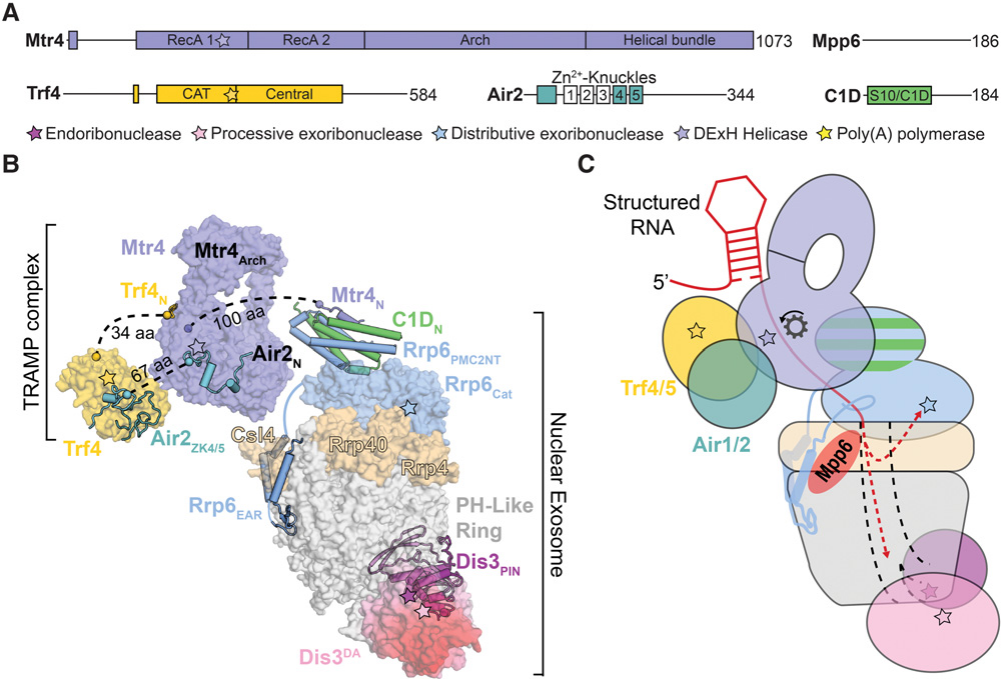
The TRAMP complex and the nuclear exosome. (*A*) Domain schematics for *S. cerevisiae* C1D, Mpp6, and TRAMP components. Catalytic sites are indicated by stars, and amino acid lengths for the *S. cerevisiae* proteins are shown. (*B*) Structural models for the nuclear exosome and associated cofactors, with RNA omitted for clarity. Black dotted lines represent connecting regions for which no structural information is available. Mtr4 and Trf4/Air2 peptides are from PDB 4U4C; Trf4/Air2 zinc knuckles are from PDB 3NYB; the PH-like ring, Rrp40, Rrp4, Csl4, Dis3, the Rrp6 catalytic module, and the Rrp6 EAR are from PDB 5K36; the Rrp6 PMC2NT domain, C1D, and the Mtr4 N-terminal peptide are from PDB 4WFD and were positioned based on PDB 5C0W. (*C*) Model for Mtr4 threading of RNA to the nuclear exosome after polyadenylation by Trf4/5. The central channel is indicated by black dashed lines, and RNA is represented as a red line with the 5′ end indicated. Dashed red arrows represent RNA paths to the catalytic subunits. Helicase direction is indicated by a gear and arrow.

**Figure 3.**
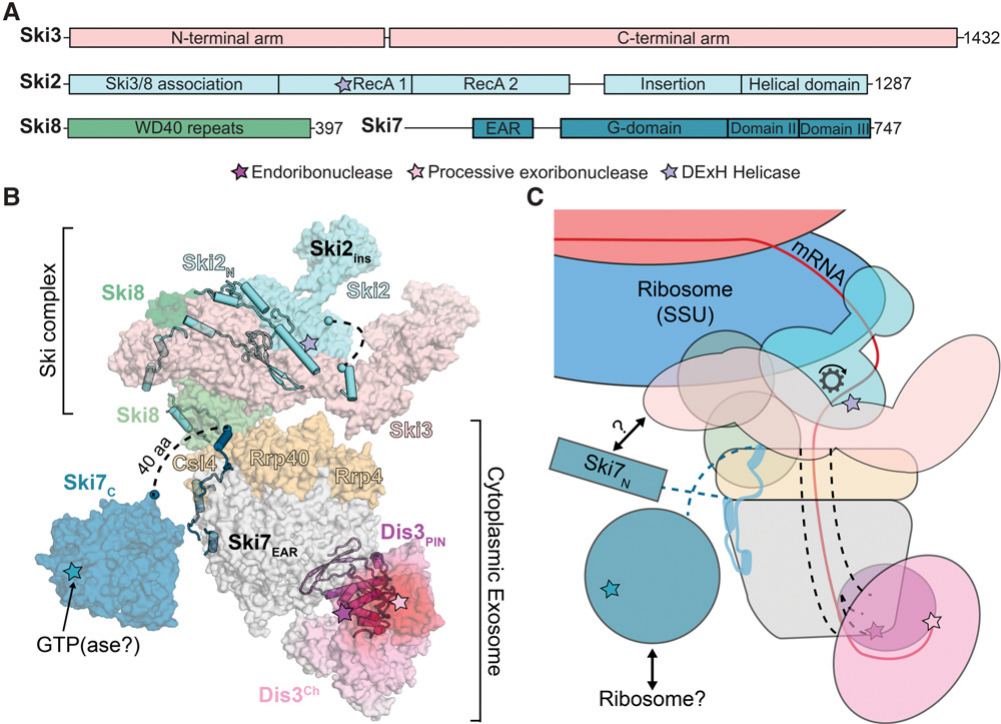
The Ski complex and the cytoplasmic exosome. (*A*) Domain schematics for *S. cerevisiae* Ski complex components. Catalytic sites are represented by stars, and amino acid lengths for the *S. cerevisiae* proteins are shown. (*B*) Structural models for the cytoplasmic exosome and associated cofactors, with RNA omitted for clarity. Black dotted lines represent connecting regions for which no structural information is available. The Ski3, Ski8, and Ski2 N termini are from PDB 4BUJ; the Ski2 globular region and insertion are from PDB 4A4Z and were aligned to PDB 4BUJ; the Ski7 CTDs are from PDB 4ZKE; the PH-like ring, Rrp40, Rrp4, Csl4, Dis3, and the Ski7 EAR are from PDB 5JEA. (*C*) Model for Ski complex channeling of a translating mRNA to the cytoplasmic exosome. The central channel is indicated by black dashed lines, and RNA is represented as a red line with the 3′ end shown bound to the Rrp44 exonuclease active site. Helicase direction is indicated by a gear and arrow.

The previous section focused on structure/activity relationships for the RNA exosome as a standalone machine; however, it is likely that exosome cofactors mediate most encounters between RNA substrates and the RNA exosome. Recent studies focused on mechanisms that recruit these cofactors to the exosome and how their various activities influence RNA decay. We restrict discussion in this section to the TRAMP and Ski complexes, as they are important and conserved modulators of RNA exosome activities in the nucleus and cytoplasm, respectively. Intact structures of TRAMP or Ski complexes bound to the exosome are not available, but structures of individual components or subcomplexes combined with genetic and biochemical studies support models as presented in Figures 2 and 3.

### The TRAMP complex

The TRAMP complex was initially uncovered by analysis of a temperature-sensitive mutant of *S. cerevisiae* that expressed hypomodified tRNA_i_^Met^ whose phenotype was suppressed by mutations in a noncanonical nuclear poly(A) polymerase (Kadaba et al. 2004). This polymerase (Trf4) and its paralog (Trf5) were found to exist in complexes with either of two zinc knuckle proteins, Air1 or Air2, and the DExH helicase Mtr4 (Fig. 2; LaCava et al. 2005; Vanacova et al. 2005; Wyers et al. 2005). TRAMP is thought to assist nuclear degradation in yeast, including decay of cryptic unstable transcripts (CUTs) and processing of rRNAs (LaCava et al. 2005; Wyers et al. 2005). The TRAMP complex genetically interacts with the RNA exosome to promote RNA degradation via its 3′ nontemplated poly(A) polymerase and RNA helicase activities and physically via interactions between the helicase and exosome (Chen et al. 2001; Schuch et al. 2014). While Mtr4 is essential and likely integrated into other complexes, an *mtr4* allele that lacks ATP-binding activity fails to rescue the lethality of a Δ*mtr4* strain (Taylor et al. 2014). With respect to TRAMP, Air1/Air2 are dispensable for viability (LaCava et al. 2005); however, simultaneous deletion of *trf4* and *trf5* results in lethality (Castaño et al. 1996). These observations underscore the importance of a functioning TRAMP complex for nuclear RNA exosome function and for viability.

Crystal structures of Mtr4 revealed a multidomain helicase core that resembles the archaeal DNA repair helicase Hel308, with a flexible insertion termed the arch domain that is specific to Mtr4 and Ski2 helicases (Fig. 2; Jackson et al. 2010; Weir et al. 2010). Biochemical and structural studies showed that Trf and Air proteins form a stable heterodimer that interacts with the helicase core of Mtr4 via short peptide motifs (Fig. 2B; LaCava et al. 2005; Hamill et al. 2010; Falk et al. 2014; Losh et al. 2015). We presume that the polyadenylation activities of TRAMP are important for generating 3′ single-stranded tails that are long enough to be captured by Mtr4, thus facilitating further unwinding to produce 3′ single-stranded tails that are long enough to be threaded into the RNA exosome central channel (Fig. 2C).

### Nuclear cofactors that bridge Mtr4 and the exosome

C1D (or Rrp47) is a small protein with functions in RNA metabolism (for review, see Mitchell 2010) and the DNA damage response (for review, see Jackson et al. 2016). While often referred to as a nuclear exosome cofactor, the recent observations that C1D is present in approximately stoichiometric amounts in endogenous nuclear complexes and is critical for stable Rrp6 expression support its inclusion as a primary subunit of the nuclear exosome (Feigenbutz et al. 2013; Shi et al. 2015). A structure of the nuclear exosome bound to C1D revealed interactions with the N-terminal PMC2NT domain of Rrp6 and its position above the Rrp6 catalytic module, forming a “lid” above the exosome (Fig. 2B; Makino et al. 2015). The composite interface between Rrp6 and C1D binds a small peptide motif near the N terminus of Mtr4, providing a physical tether to the exosome (Fig. 2B; Schuch et al. 2014), while the C-terminal region of C1D interacts with protein components of box C/D snoRNPs (Costello et al. 2011). With that said, the NTD of C1D fully rescues growth in synthetic-lethal Δ*rex1*Δ*rrp47* and Δ*mpp6*Δ*rrp47 S. cerevisiae* strains, suggesting that the most critical functions for C1D may pertain to Mtr4/TRAMP recruitment and stabilization of Rrp6 (Costello et al. 2011; Garland et al. 2013).

Mpp6 is another small, nucleic acid-binding protein that associates with the Exo9 core; however, it is estimated to be present in 10% of nuclear complexes in *S. cerevisiae* (Schilders 2005; Schuch et al. 2014; Shi et al. 2015). Cross-linking experiments coupled to mass spectrometry showed that it contacts the S1/KH protein Rrp40 in the yeast complex (Shi et al. 2015), and competition binding experiments at high concentrations reported low-affinity interactions between its C-terminal region and the Rrp6 catalytic domain (Kim et al. 2016). Human Mpp6 interacts directly with Mtr4 (Chen et al. 2001), supporting a role in recruitment of Mtr4 or Mtr4 complexes in higher eukaryotes. This role may also be conserved in *S. cerevisiae*, as interactions between the N terminus of Mtr4 and the Rrp6/C1D lid are relatively weak (Schuch et al. 2014).

### The Ski complex

The cytoplasmic Ski complex consists of the DExH-box helicase Ski2, the tetratricopeptide repeat scaffold protein Ski3, and two copies of the β-propeller protein Ski8p (Fig. 3; Brown et al. 2000; van Hoof et al. 2000; Halbach et al. 2013). The Ski complex contributes to mRNA turnover, degradation of aberrant mRNAs, viral defense, and RNAi pathways in some eukaryotes (for review, see Łabno et al. 2016). Deletion of any subunit of the Ski complex in *S. cerevisiae* results in synthetic lethality when combined with mutations of decapping enzymes or deletion of the 5′-to-3′ exoribonuclease Xrn1 (Anderson and Parker 1998; van Hoof et al. 2000; Araki et al. 2001). Ski2 contains an N-terminal region that is necessary for Ski complex interactions followed by a helicase core and a flexible insertion domain, similar to features observed in Mtr4 (Fig. 3; Wang et al. 2005; Halbach et al. 2012, 2013). Association of the Ski complex with the exosome extends the through-channel RNA path to Dis3 by ∼10 nt, leading to a model for channeling (Fig. 3C; Halbach et al. 2013). A recent cryo-EM structure of the Ski complex bound to the ribosome translating an mRNA with a 3′ overhang showed that the Ski complex binds the small subunit (SSU) of the ribosome via interactions between the Ski2 insertion and helicase core domains and one of the Ski8 protomers (Fig. 3C; Schmidt et al. 2016).

### Ski7/HBS1Lv3 bridges the Ski complex and RNA exosome

Yeast Ski7 contains a globular C-terminal GTPase domain that is proposed to interact with the ribosome and NTDs that bridge the RNA exosome and Ski complexes (Araki et al. 2001; van Hoof et al. 2002; Wang et al. 2005). A crystal structure of the C-terminal module of *S. cerevisiae* Ski7 revealed its structural similarity to active translational GTPases, although GTPase activity could not be confirmed in vitro (Kowalinski et al. 2015). Aligned two-dimensional (2D) class averages from cryo-EM analysis of endogenous *S. cerevisiae* Ski7-containing cytoplasmic exosome complexes suggest that the C-terminal globular domains of Ski7 adopt multiple conformations when bound to the exosome, and three-dimensional (3D) reconstructions revealed that the Ski7 NTD interacts with the Exo9 core via surfaces that overlap with those used by the Rrp6 CTD (Liu et al. 2016). This latter result was confirmed with binding assays and observed in a contemporaneous crystal structure (Kowalinski et al. 2016; Liu et al. 2016). Additionally, two groups independently identified a short splicing isoform of human HBS1L (HBS1Lv3) as the long sought after Ski7 homolog and confirmed its ability to interact with the human exosome and Ski complex (Kalisiak et al. 2016; Kowalinski et al. 2016). The canonical HBS1L isoform in humans contains a C-terminal GTPase fold that the exosome-interacting isoform lacks but does not interact with the exosome, suggesting that multiple subcomplexes of the Ski complex may exist in higher eukaryotes.

## Targeting RNAs to the exosome and associated complexes

The RNA exosome can cooperate with its cofactors to specifically target transcripts for degradation. Selective targeting has reported roles in diverse processes such as heterochromatic silencing, suppression of untimely meiosis in *Schizosaccharomyces pombe*, and viral defense (Harigaya et al. 2006; Lee et al. 2013; Aly et al. 2016; Molleston et al. 2016). Since targeting of RNA for degradation by the exosome based on RNA sequence elements was covered in a recent review (Kilchert et al. 2016), we discuss alternative mechanisms of RNA targeting in this final section.

### Cofactor-mediated bridging to the cap-binding complex

In addition to TRAMP, human MTR4 (also called SKIV2L2) is present in at least two other complexes, the nuclear exosome targeting (NEXT) and poly(A) tail exosome targeting (PAXT) complexes (Lubas et al. 2011; Meola et al. 2016). These complexes include mutually exclusive interactions between MTR4, a zinc finger protein (ZCCHC8 in NEXT and ZFC3H1 in PAXT), and a RNA-binding protein (RBM7 in NEXT and PABPN1 in PAXT). The NEXT complex promotes degradation of PROMPTs and 3′ extended RNAs, including aberrant hTR, in the nucleoplasm (Preker et al. 2008; Lubas et al. 2011; Tseng et al. 2015). A recent study showed that ZCCHC8 interacts with the RNA recognition motif (RRM) domain of RBM7 via a proline-rich sequence and bridges interactions with MTR4 in the complex (Falk et al. 2016). PAXT promotes degradation of longer and more mature [with respect to poly(A) tail length] substrates compared with NEXT substrates (Meola et al. 2016). Both NEXT and PAXT interact with the cap-binding complex containing ARS2 (CBCA complex) via an adaptor protein, ZC3H18, physically tethering the exosome to nascent capped transcripts to promote degradation following termination (Andersen et al. 2013; Meola et al. 2016).

In *S. pombe*, a nuclear Mtr4-like helicase, Mtl1, associates with a zinc finger protein, Red1, to form the MTREC core complex, which is involved in heterochromatic silencing and degradation of meiotic RNAs, CUTs, and unspliced transcripts (Lee et al. 2013; Egan et al. 2014; Zhou et al. 2015). MTREC also interacts with the CBCA complex and the exosome, and the Red1 subunit shares homology with ZFC3H1 (Lee et al. 2013; Zhou et al. 2015), possibly indicating that it is the fission yeast counterpart to the human PAXT complex. Importantly, NEXT, PAXT, and MTREC complexes lack 3′ polyadenylation activity, suggesting that their RNA-binding and helicase activities are sufficient to generate 3′ ssRNA that is long enough to engage the exosome.

### Mtr4/AIM interactions for selective RNA decay

**Figure 4.**
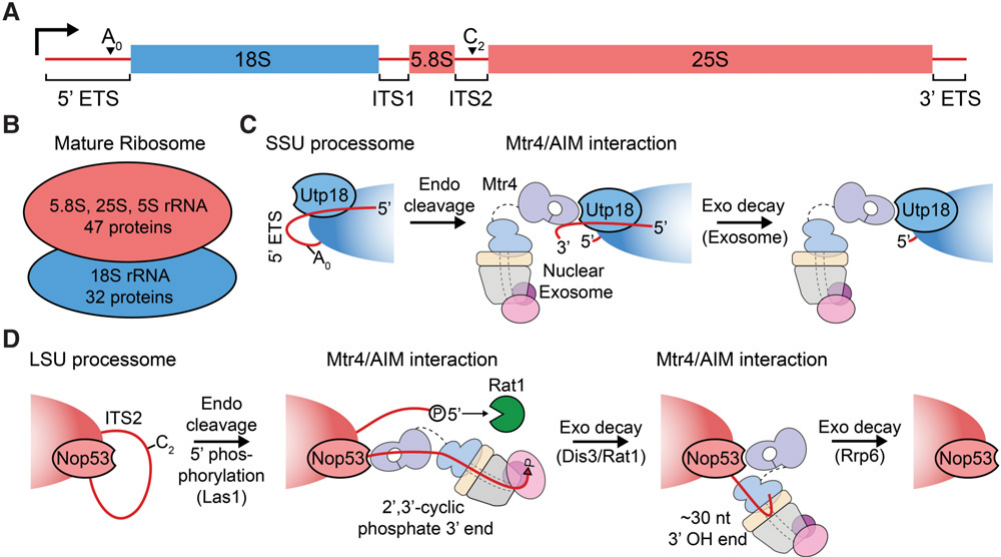
AIM–Arch interactions recruit the exosome for rRNA processing (*A*) Schematic for the *S. cerevisiae* 7-kb pre-rRNA molecule. Regions of the RNA contained within the mature ribosome are shown as boxes, and spacers are shown as lines. The direction of transcription is shown with an arrow, and A_0_ and C_2_ endonucleolytic cleavage sites are indicated. (*B*) Schematic for a mature ribosome. (*C*) A Utp18_AIM_–Mtr4_Arch_ interaction recruits the exosome for 5′ externally transcribed spacer (ETS) removal after endonucleolytic cleavage at the A_0_ site. (*D*) A Nop53_AIM_–Mtr4_Arch_ interaction recruits the exosome for 5.8S rRNA processing after Las1 cleavage at the C_2_ site.

rRNAs are derived from two transcripts in *S. cerevisiae*: one that codes for the 18S, 5.8S, and 25S rRNAs and another that codes for the 5S rRNA (Fig. 4A,B; for review, see Henras et al. 2014). A set of proteins known as the processome dynamically associates with the pre-rRNA cotranscriptionally to direct endonucleolytic and exonucleolytic RNA processing in addition to chaperoning ribosomal proteins within the RNP (for review, see Woolford and Baserga 2013). By aptamer tagging and affinity-purifying rRNAs of different lengths, a recent study dissected the order in which processome subcomplexes associate with and dissociate from the pre-rRNA that encompasses the SSU (Chaker-Margot et al. 2015), although molecular details underlying these interactions remain elusive.

Another recent study addressing rRNA processing uncovered a conserved motif (arch-interacting motif [AIM]) in processome proteins Utp18 and Nop53 that interacts with the arch of Mtr4 to recruit the exosome (Thoms et al. 2015). They found that interaction of the Mtr4 arch with this motif in Utp18, a subunit of the early associating processome subcomplex UtpB, enables removal of the 5′ externally transcribed spacer (ETS), while interaction with Nop53, a later associating processome factor, enables processing of the 5.8S RNA (Fig. 4C,D). A cryo-EM-derived model of the SSU processome at 5.1-Å resolution revealed that the β-propeller domain of Utp18 resides on the periphery of the complex ∼75 Å from the A_0_ site of the 5′ ETS, although its N-terminal ∼250 amino acids (which contain the AIM) could not be modeled (Fig. 4C; Chaker-Margot et al. 2016). Mutation of the AIM on Utp18 or Nop53 resulted in accumulation of unprocessed precursors for their respective substrates. Interestingly, other proteins such as Air2 and Sqs1 can interact with the Mtr4 arch in a region similar to Nop53/Utp18, suggesting that hierarchical competition for the arch may contribute to RNA decay in the nucleus (Losh and van Hoof 2015; Thoms et al. 2015).

### 3′ end chemistry and RNA fate

Dis3- or Rrp6-mediated degradation of model substrates by exosomes containing Dis3 and Rrp6 appears stochastic in vitro; however, this seems an unlikely strategy for selective targeting of RNA for processing or degradation in vivo. While nuclear cofactor complexes can influence RNA fate as discussed, recent studies suggested that the chemical structure of the 3′ end might influence the fate of a particular RNA. For example, Rrp6 from humans and *S. cerevisiae* lacks activity on synthetic RNA containing a 3′ phosphate, while Dis3 family enzymes readily degrade these RNAs (Burkard and Butler 2000; Tomecki et al. 2010; Lubas et al. 2013; Domanski et al. 2016; Zinder et al. 2016). While RNA with 3′ phosphate may not be prevalent in vivo, 3′ phosphate ends can result from random breaks elicited by damaging agents or by purposeful endonucleolytic cleavage. Examples of the latter include 2′,3′-cyclic phosphate modification, the product of maturation reactions for a number of RNAs, including tRNA introns, U6 snRNA, and rRNA precursors (Knapp et al. 1979; Lund and Dahlberg 1992; Gasse et al. 2015). Although untested, structural studies suggest that Rrp44, but not Rrp6, could degrade RNAs with 2′,3′-cyclic phosphate 3′ ends (Zinder et al. 2016).

For U6 RNA, Mpn1 (also called Usb1) generates a 2′,3′-cyclic phosphate at the 3′ end as a product of its exonuclease activity, and U6 is polyadenylated and rapidly degraded in a Δ*mpn1 S. pombe* strain (Shchepachev et al. 2012, 2015). This suggests that a 2′,3′-cyclic phosphate stabilizes U6 by preventing decay by Rrp6 or polyadenylation by TRAMP. Indeed, a transcriptome-wide survey of 2′,3′-cyclic phosphate RNAs revealed that U6 is by far the most abundant RNA with this chemical signature in HeLa cells, although this approach may have missed other RNAs that are targeted for rapid degradation (Schutz et al. 2010).

To generate the 7S rRNA (5.8S rRNA with a 3′ extension), the Las1 endonuclease component of the Las1 complex cleaves the rRNA precursor molecule at the C_2_ site of internally transcribed spacer 2 (ITS2) (Fig. 4A; Gasse et al. 2015), resulting in a 2′,3′-cyclic phosphate at the 3′ end of the 7S rRNA and a 5′OH on the other fragment. While the latter RNA is 5′ phosphorylated by the Las1 complex to enable processing by the nuclear 5′-to-3′ exonuclease Rat1/Xrn2 (Fig. 4D), the 2′,3′-cyclic phosphate end is an intermediate prior to processive 3′-to-5′ processing by Dis3 (Fig. 4D). This produces the 5.8S rRNA plus ∼30 nt at the 3′ end, the approximate length required to span the Exo9 central channel. This overhang is subsequently removed by Rrp6 (Fig. 4D; Briggs et al. 1998; Allmang et al. 1999a). While this and other 2′,3′-cyclic phosphate 3′ ends may be resolved by phosphodiesterases prior to processing or degradation, it remains possible that 3′-modified RNAs could be substrates of Dis3 and the nuclear RNA exosome.

## Conclusions and future challenges

The RNA exosome and its cofactors provide a versatile platform for targeting a wide variety of RNA substrates for processing and/or degradation in the nucleus and cytoplasm. Although the exosome was discovered two decades ago, it seems that we are just beginning to piece together its complexity. Armed with various cofactors that presumably recruit and/or localize the exosome to different subcellular compartments or complexes, it remains unclear how particular complexes compete with one another to promote these functions. With respect to structural studies, it remains a significant challenge to capture intermediates during recruitment and/or degradation, as most factors require a 3′ end for activity. For instance, TRAMP-mediated decay includes at least four enzymes that bind an RNA 3′ end: a 3′ nontemplated poly(A) polymerase, a 3′-to-5′ RNA helicase, and two 3′-to-5′-directed exoribonucleases (Fig. 2). Perhaps most relevant is the need to uncover regulatory mechanisms that mark a particular RNA for degradation. In this context, we are again reminded of conceptual parallels between the RNA exosome and ubiquitin–proteasome pathways, beyond those noted previously. While the proteasome was long viewed as a trash can for protein degradation, the discovery of a vast network of regulated processes involving ubiquitin E2-conjugating enzymes, E3 ligases, and shuttling factors that deliver substrates to the proteasome made it clear that protein degradation was not a passive process but an active and highly regulated quality control pathway. Many cofactors for the exosome have been uncovered; however, regulatory signals underlying their activities and/or specificities will remain an area of interest for years to come.
